# Supplementary material for: Heparin-based hydrogel scaffolding alters the transcriptomic profile and increases the chemoresistance of MDA-MB-231 triple-negative breast cancer cells
Source: Biomater Sci. 2020 Feb 13;8(10):2786–96. doi: 10.1039/c9bm01481k (PMC7497406; doi:10.1039/c9bm01481k)
Supplement: Supplementary file 2 [file BM-008-C9BM01481K-s002.zip › Supplementary File 4/HP-B hydrogel + EGF (s)vControl/Pathways/my_analysis.Gsea.1545201170987/gsea_report_for_CONTROL_1545201170987.html]

Report for CONTROL 1545201170987 [GSEA]

| GS  follow link to MSigDB | GS DETAILS | SIZE | ES | NES | NOM p-val | FDR q-val | FWER p-val | RANK AT MAX | LEADING EDGE || 1 | HALLMARK\_OXIDATIVE\_PHOSPHORYLATION | Details ... | 194 | -0.60 | -2.89 | 0.000 | 0.000 | 0.000 | 3893 | tags=56%, list=20%, signal=69% |
| 2 | HALLMARK\_PROTEIN\_SECRETION | Details ... | 94 | -0.60 | -2.59 | 0.000 | 0.000 | 0.000 | 3368 | tags=52%, list=18%, signal=63% |
| 3 | HALLMARK\_COAGULATION | Details ... | 87 | -0.60 | -2.53 | 0.000 | 0.000 | 0.000 | 3605 | tags=53%, list=19%, signal=65% |
| 4 | HALLMARK\_INTERFERON\_GAMMA\_RESPONSE | Details ... | 167 | -0.51 | -2.38 | 0.000 | 0.000 | 0.000 | 3161 | tags=40%, list=16%, signal=47% |
| 5 | HALLMARK\_INTERFERON\_ALPHA\_RESPONSE | Details ... | 91 | -0.53 | -2.26 | 0.000 | 0.000 | 0.000 | 3161 | tags=40%, list=16%, signal=47% |
| 6 | HALLMARK\_HYPOXIA | Details ... | 163 | -0.48 | -2.26 | 0.000 | 0.000 | 0.000 | 2997 | tags=39%, list=16%, signal=45% |
| 7 | HALLMARK\_ANDROGEN\_RESPONSE | Details ... | 91 | -0.52 | -2.23 | 0.000 | 0.000 | 0.000 | 3436 | tags=45%, list=18%, signal=55% |
| 8 | HALLMARK\_COMPLEMENT | Details ... | 141 | -0.49 | -2.22 | 0.000 | 0.000 | 0.000 | 3569 | tags=43%, list=19%, signal=53% |
| 9 | HALLMARK\_ALLOGRAFT\_REJECTION | Details ... | 116 | -0.49 | -2.16 | 0.000 | 0.000 | 0.000 | 3201 | tags=40%, list=17%, signal=47% |
| 10 | HALLMARK\_IL6\_JAK\_STAT3\_SIGNALING | Details ... | 63 | -0.54 | -2.15 | 0.000 | 0.000 | 0.000 | 3184 | tags=40%, list=17%, signal=47% |
| 11 | HALLMARK\_GLYCOLYSIS | Details ... | 171 | -0.45 | -2.14 | 0.000 | 0.000 | 0.000 | 3443 | tags=37%, list=18%, signal=44% |
| 12 | HALLMARK\_MTORC1\_SIGNALING | Details ... | 190 | -0.44 | -2.10 | 0.000 | 0.000 | 0.000 | 4037 | tags=44%, list=21%, signal=55% |
| 13 | HALLMARK\_ADIPOGENESIS | Details ... | 176 | -0.44 | -2.09 | 0.000 | 0.000 | 0.000 | 3679 | tags=40%, list=19%, signal=49% |
| 14 | HALLMARK\_KRAS\_SIGNALING\_UP | Details ... | 144 | -0.45 | -2.06 | 0.000 | 0.000 | 0.000 | 4555 | tags=46%, list=24%, signal=60% |
| 15 | HALLMARK\_FATTY\_ACID\_METABOLISM | Details ... | 131 | -0.43 | -1.99 | 0.000 | 0.000 | 0.002 | 4984 | tags=52%, list=26%, signal=70% |
| 16 | HALLMARK\_APOPTOSIS | Details ... | 140 | -0.43 | -1.96 | 0.000 | 0.000 | 0.002 | 2921 | tags=36%, list=15%, signal=43% |
| 17 | HALLMARK\_EPITHELIAL\_MESENCHYMAL\_TRANSITION | Details ... | 150 | -0.42 | -1.95 | 0.000 | 0.000 | 0.002 | 2653 | tags=34%, list=14%, signal=39% |
| 18 | HALLMARK\_MYC\_TARGETS\_V1 | Details ... | 189 | -0.40 | -1.92 | 0.000 | 0.001 | 0.005 | 4422 | tags=43%, list=23%, signal=56% |
| 19 | HALLMARK\_PEROXISOME | Details ... | 85 | -0.45 | -1.91 | 0.000 | 0.001 | 0.005 | 3216 | tags=36%, list=17%, signal=44% |
| 20 | HALLMARK\_REACTIVE\_OXIGEN\_SPECIES\_PATHWAY | Details ... | 44 | -0.50 | -1.89 | 0.000 | 0.001 | 0.005 | 2611 | tags=39%, list=14%, signal=45% |
| 21 | HALLMARK\_BILE\_ACID\_METABOLISM | Details ... | 80 | -0.44 | -1.85 | 0.000 | 0.001 | 0.007 | 3214 | tags=36%, list=17%, signal=43% |
| 22 | HALLMARK\_TNFA\_SIGNALING\_VIA\_NFKB | Details ... | 178 | -0.38 | -1.82 | 0.000 | 0.001 | 0.011 | 4530 | tags=47%, list=24%, signal=61% |
| 23 | HALLMARK\_IL2\_STAT5\_SIGNALING | Details ... | 155 | -0.39 | -1.79 | 0.000 | 0.001 | 0.013 | 3350 | tags=32%, list=17%, signal=39% |
| 24 | HALLMARK\_INFLAMMATORY\_RESPONSE | Details ... | 130 | -0.40 | -1.79 | 0.000 | 0.001 | 0.013 | 4739 | tags=48%, list=25%, signal=63% |
| 25 | HALLMARK\_CHOLESTEROL\_HOMEOSTASIS | Details ... | 67 | -0.44 | -1.76 | 0.002 | 0.002 | 0.021 | 3322 | tags=36%, list=17%, signal=43% |
| 26 | HALLMARK\_HEME\_METABOLISM | Details ... | 166 | -0.35 | -1.67 | 0.000 | 0.005 | 0.059 | 3411 | tags=32%, list=18%, signal=38% |
| 27 | HALLMARK\_ESTROGEN\_RESPONSE\_LATE | Details ... | 161 | -0.33 | -1.52 | 0.000 | 0.016 | 0.191 | 3361 | tags=27%, list=18%, signal=32% |
| 28 | HALLMARK\_XENOBIOTIC\_METABOLISM | Details ... | 151 | -0.31 | -1.43 | 0.000 | 0.033 | 0.363 | 4521 | tags=37%, list=24%, signal=48% |
| 29 | HALLMARK\_PI3K\_AKT\_MTOR\_SIGNALING | Details ... | 89 | -0.32 | -1.38 | 0.020 | 0.046 | 0.490 | 3523 | tags=34%, list=18%, signal=41% |
| 30 | HALLMARK\_PANCREAS\_BETA\_CELLS | Details ... | 18 | -0.47 | -1.38 | 0.090 | 0.045 | 0.491 | 4572 | tags=50%, list=24%, signal=66% |
| 31 | HALLMARK\_P53\_PATHWAY | Details ... | 179 | -0.28 | -1.32 | 0.028 | 0.066 | 0.642 | 2885 | tags=23%, list=15%, signal=27% |
| 32 | HALLMARK\_ESTROGEN\_RESPONSE\_EARLY | Details ... | 170 | -0.27 | -1.26 | 0.044 | 0.103 | 0.801 | 3151 | tags=23%, list=16%, signal=27% |
| 33 | HALLMARK\_DNA\_REPAIR | Details ... | 139 | -0.26 | -1.19 | 0.106 | 0.165 | 0.936 | 4375 | tags=32%, list=23%, signal=42% |
| 34 | HALLMARK\_ANGIOGENESIS | Details ... | 23 | -0.37 | -1.19 | 0.220 | 0.167 | 0.944 | 3102 | tags=35%, list=16%, signal=41% |
| 35 | HALLMARK\_APICAL\_JUNCTION | Details ... | 147 | -0.23 | -1.05 | 0.333 | 0.392 | 1.000 | 2866 | tags=20%, list=15%, signal=23% |
| 36 | HALLMARK\_UNFOLDED\_PROTEIN\_RESPONSE | Details ... | 105 | -0.23 | -0.99 | 0.424 | 0.533 | 1.000 | 4093 | tags=29%, list=21%, signal=36% |
| 37 | HALLMARK\_UV\_RESPONSE\_UP | Details ... | 131 | -0.22 | -0.99 | 0.461 | 0.528 | 1.000 | 4579 | tags=35%, list=24%, signal=46% |
| 38 | HALLMARK\_HEDGEHOG\_SIGNALING | Details ... | 26 | -0.30 | -0.97 | 0.486 | 0.574 | 1.000 | 1921 | tags=15%, list=10%, signal=17% |
| 39 | HALLMARK\_APICAL\_SURFACE | Details ... | 29 | -0.29 | -0.96 | 0.542 | 0.583 | 1.000 | 3679 | tags=38%, list=19%, signal=47% |
| 40 | HALLMARK\_NOTCH\_SIGNALING | Details ... | 28 | -0.27 | -0.89 | 0.603 | 0.751 | 1.000 | 3985 | tags=39%, list=21%, signal=50% |
| 41 | HALLMARK\_UV\_RESPONSE\_DN | Details ... | 129 | -0.17 | -0.77 | 0.966 | 0.936 | 1.000 | 3661 | tags=20%, list=19%, signal=25% |
Table: Gene sets enriched in phenotype **CONTROL (2 samples) **[plain text format]****
